# DEVELOPING AND TESTING AN ASSISTED LIVING ENVIRONMENT MODEL

**DOI:** 10.1093/geroni/igz038.2374

**Published:** 2019-11-08

**Authors:** Sarah D Holmes, Elizabeth Galik, Barbara Resnick

**Affiliations:** 1 University of Maryland Baltimore Doctoral Program in Gerontology, Baltimore, Maryland, United States; 2 University of Maryland School of Nursing, Baltimore, Maryland, United States

## Abstract

The assisted living (AL) environment plays an important role in supporting residents’ life satisfaction and helping them to age in place. Guided by ecological theory, the AL environment is multidimensional and has many interrelated components including staffing (e.g. direct care workers, nursing, activity staff), services provided (e.g. medical, mental health, pharmacy), amenities offered at the setting (e.g. beauty salon, computer room, exercise facilities), and built environment features (e.g. walkability). Moreover, evidence suggests that aspects of the AL environment can enhance or detract from the physical function, well-being, social engagement, and behavioral outcomes among residents. The purpose of this study was to develop and test an integrative AL environment measurement model that includes indicators of staffing, services, amenities, and the built environment. Baseline data was used from a study testing the Dissemination and Implementation of Function Focused Care in AL. A total of 54 AL facilities across three states were included in the sample. Settings ranged in size from 31 to 164 beds with an average size of 82.2 (SD=26.2) beds and the majority were for profit facilities (n=41, 74.5%). Structural equation modeling was used to test the proposed model. Results showed that the model fit the data (chi-squared/df=1.86, p<.05; CFI=.858, RMSEA=.126). Having an integrative AL environment measurement model will advance future research that explores the impact of the environment on resident outcomes. In addition, findings from this study can inform interventions and programs designed to modify AL environments to optimize residents’ ability to age in place.

